# Assessing Ethoshunt as a Gamification-Based Mobile App in Ethics Education: Pilot Mixed-Methods Study

**DOI:** 10.2196/18247

**Published:** 2020-08-10

**Authors:** Noor Syamilah Zakaria, M Iqbal Saripan, Neerushah Subarimaniam, Alyani Ismail

**Affiliations:** 1 Department of Counselor Education and Counseling Psychology Faculty of Educational Studies Universiti Putra Malaysia Selangor Darul Ehsan Malaysia; 2 Department of Computer and Communication Systems Engineering Faculty of Engineering Universiti Putra Malaysia Selangor Darul Ehsan Malaysia

**Keywords:** gamification, ethics, education, ethics education, ethical competency, mobile app, mobile app usability

## Abstract

**Background:**

Gamification has remarkable potential in the learning space. The process of creating a gamified system and its influence on human behavior reflect the interaction between educators and machines.

**Objective:**

The purpose of this pilot study was to present Ethoshunt as a gamification-based mobile app that can be used in teaching and learning ethics.

**Methods:**

This study involved a mixed-methods research design. The researchers surveyed 39 undergraduate students who were introduced to Ethoshunt in order to examine the relationships between mobile app usability and positive emotions, ethical competency, and user experience. Affinity diagramming was used as a tool to organize the opinions and experiences of participants using featured gamification elements.

**Results:**

Game dynamics and game mechanics explained the functionality of Ethoshunt. In addition, the learning flow through Ethoshunt was discussed. Overall, the findings were positive, and mobile app usability had the strongest relationship with positive emotions (*r*=0.744, *P*<.001), followed by ethical competency (*r*=0.686, *P*<.001) and user experience (*r*=0.614, *P*<.001).

**Conclusions:**

Positive emotions could be perceived as an important dimension in the development and usability of Ethoshunt. The researchers suggest that the gamification-based mobile app advocated in this study may provide ideas for ethics educators who wish to develop a technology-mediated learning environment.

## Introduction

### Background

Gamification is the use of a game-like concept to create engaging and supportive learning environments that motivate students. Elements of games have been historically important in teaching and learning. Game elements effectively engage students, and the primary role of educators to engage students in learning is becoming a priority [1-3]. According to the United Nations Educational, Scientific, and Cultural Organization (UNESCO), the process of integrating new tools and technologies that involve game elements depends on the educator’s ability to: (1) construct a learning environment by employing new ideas; (2) consolidate new technology with a new pedagogy; and (3) promote socially active classrooms to encourage group work, cooperative interaction, and collaborative learning [4]. Furthermore, integrating game elements in education is a technique that is used to foster collaboration among students [5] and to extend learning while developing their cognitive, emotional, and social dimensions [6].

The application of game elements, such as game dynamics and game mechanics, commonly known as “gamification” has been entering the realms of education, lifestyle, and health [7]. In the education context, gamification is regarded as a tool that has been influential [8] and a prominent trend in recent years [9]. It creates a great deal of value for students since it encourages learning through entertainment [10]. With multifunction tools, gamification is defined as the use of game design elements in nongame contexts [11]. The entire process of gamification is to create interactions between educators and machines, but not to replace educators [11].

Gamification is an application that integrates both game mechanics and game dynamics with the support of a virtual learning environment, offering various benefits to humans [12]. Previous studies have investigated the development and usage of gamification across disciplines. For instance, gamification positively influences consumers’ behaviors in switching intention between membership cards and mobile apps [13]. On the other hand, gamification increases bicycle riding activities and logging behaviors through a point system [14], promotes the quality of teacher education and in-service training, assists researchers in environmental multicriteria decision analysis specifically related to water [15], and supports collaborative learning in primary education through implementation of a tactile and tangible multitablet gamified quiz system [2].

Gamification researchers have also explored personality types that can predict players’ preferences for game elements and mechanics [7], as well as energy-related behaviors that are necessary to engage residential customers in energy application enabled by smart meters [16]. Interestingly, many researchers have designed gamification-based applications to capture consumers’ fun and “gameful” experiences [9] and to observe weight loss in overweight and obese adolescents, which eventually improves their health conditions [12]. On the other hand, Zhang et al [17] used gamification for cognitive bias modification. Besides usage, usability of gamification is equally important as it enables users to perform tasks effectively and efficiently. Usability ensures a gamification-based tool impacts users in many ways, such as achieving goals, increasing proactive behaviors, and improving performance; otherwise, the tool would be worthless.

Similarly, usability was the main concern in this study. It is critically important to measure the usability of a gamification-based tool to ensure it is usable, useful, and effective in teaching and learning ethics. Therefore, the aim of this study was to present the development of a gamification-based mobile app called Ethoshunt and assessment of its usability in ethics education. The researchers used a correlation method to examine the relationships between mobile app usability and positive emotions, ethical competency, and user experience. Opinions from students based on their experiences of using Ethoshunt were also gathered. The results were expected to provide evidence of the effectiveness and usability of Ethoshunt.

### Related Work

#### Gamification in Ethics Education

Gamification has a large potential in teaching and learning activities. Furthermore, gamification strengthens teaching and learning processes [18]. Through gamification, students as gamification-based application users potentially exhibit positive learning behaviors. In addition, gamification is almost everywhere in the form of puzzles; adventure, simulation, edutainment, and strategy games; and real-time strategy games [19]. Gamification reflects a shifting idea of using game design elements, such as the mechanics and dynamics of games, in nongame themes, products, contexts, and services to make difficult tasks easier [10].

Gamification is an effective engagement tool that reinforces human behaviors through specific educational activities with the assistance of game mechanics, such as levels, ranks, missions, and instant rewards [20]. The most used game mechanics are badges, points, levels, prizes, storytelling, progress bars, and leaderboards [21-23]. For instance, an ethics educator may not conduct formative assessments in tutorial classes, and instead, may replace it with gamification-based assessments inclusive of prizes that would create game-based experiences in educational contexts. These experiences would increase students’ relatedness, competence, and autonomy [19,24]. Furthermore, gamification is pleasing, engaging [17,25], and sustainable, as it provides an interactive educational environment. Education-based gamification systems, such as GamiCad, Jigsaw, and Gamified Multimedia Content Production, guarantee students’ quick task completion during the process of reviewing the lessons learned [26].

Gamification allows students to experience a feeling of energized focus, enjoy the process of classroom activity, and get involved in the learning process [2]. In addition, gamification can be utilized as a motivational tool [1,8,27], promotes a flexible learning environment, and provides challenges to high-ability students [12]. In addition, students can maintain positive attitudes toward courses integrated with gamification, and gamification can influence students’ learning and achievements [28]. Students are generally enthusiastic about gamification and learn more with gamification. The previous findings reflect that nontraditional teaching methods integrating technology-mediated learning can enhance students’ engagement in the classroom. However, it is important to recruit quality educators, as they are expected to make efficient use of technical infrastructure, use technology effectively, enable effective communication, adapt gamification content to the ethics course, and interpret the outcome of gamification-based applications [29].

An alternative concept to build a motivating nature in game-based pedagogy is ethics educators’ competencies. Besides effectively making use of the technology, ethics educators are expected to be competent in four areas, including pedagogical, collaborative, technological, and creative areas [20]. A pedagogically competent educator ought to have the ability to engage students in planning and designing gamification, requiring collaboration (second area) within the school and other schools. Moreover, educators must be technologically competent, as they will need to analyze games and technological tools. It is expected as well that technologically competent educators will be able to overcome technology-related obstacles [20]. Finally, creative educators are needed to create playful learning zones to explore and enhance gamification in education. The creativity aspect of teaching would assist in sustaining students’ motivation and promote self-development. For the motivation aspect, gamification promotes behavioral changes. Timpel et al [12] mentioned that motivation is the heart of gamification. Gamification works best to boost motivation through engagement, which can make the most challenging and tedious tasks enjoyable [27]. Timpel et al [12] further revealed that the aspect of motivation in gamification can be explained further according to the self-determination theory and organismic integration theory.

The self-determination theory focuses on what drives students to make choices without external influences and argues that students or gamification users are self-motivated and self-determined as the gamified system provides them a sense of social relatedness and autonomy [12]. The elements in gamification ultimately create a state of intrinsic motivation that influences users to go through challenges and make achievements. According to Su and Cheng [22], intrinsic motivation that is developed based on feelings of competence and autonomy will keep students engaged and help them learn actively for many hours with no rewards. On the other hand, the organismic integration theory describes different types of extrinsic motivation and highlights that behavioral regulations are experienced as comparatively alien to the self [30]. In other words, individuals’ actions are compelled by externally controlled rewards or punishments. For example, students who are exposed to gamification in conservative classroom lectures will feel motivated, specifically when they are rewarded with progress bars, points, or badges, and the motivation indirectly builds collaboration with other learning peers.

Gamification has the potential to transform an extrinsic experience into internal meaning and help students to reflect upon and reorganize their familiarities [31,32]. However, in the long run, the reward mechanism, which also serves as a controlling aspect, may erode intrinsic motivation [12]. Thus, it is essential to select meaningful educational game elements that are in line with the users’ interests and goals to sustain their intrinsic motivation. According to Rodrigues et al [2], points, quizzes, and challenges are the most used game elements.

#### Assessing Positive Emotions, Ethical Competency, User Experience, and Mobile App Usability

Positive emotions refer to positive effects functioning as internal signals that increase the motivation level [33]. In addition, positive emotions, such as joy, contentment, love, and interest, are markers of optimal well-being or flourishing. Positive emotions are recognizable via vocal, visual, auditory, touch, and facial cues [34]. In the context of this study, gamification has a tendency to provide positive emotions that may break existing habits and substitute the habitual behaviors with new stabilized behaviors [35]. Gamification invokes students’ behaviors in the classroom by activating their motivation [36] and sustaining focus in the classroom. For example, students who are exposed to gamification in the classroom can learn in a fun environment while sharing their experiences with educators and learning peers. The game dynamics and game mechanics in gamification-based tools attract students, as they increase students’ positive emotions, which rationally makes them invest more time in exploring the game, boosts motivation, and fosters their curiosity to know more about the game. According to Korn et al [36], gamification is able to shift the students’ mood spectrum toward a more relaxed condition. The delightful concept of gamification involves more emotions; however, the happy and unhappy emotions balance each other [36].

Ethical competency refers to ethical behaviors and actions that require ethical knowledge and reflection [37]. Ethical competency aids in achieving organizational goals, improves individuals’ performances, improves quality of the services provided, and invites positive consequences to the organization [38]. Ethical competency is critical to the well-being of students. Living ethically can be articulated as projecting the highest standard of the belief system. To comprehensively teach ethics education in a semester is a challenge; nonetheless, a foundation of knowledge, thinking, and acting can be set in place [26,32,39,40]. In this study, the ethics education instructor used gamification for disseminating knowledge from the literature and text books, dialoguing in conservative classroom lectures regarding knowledge application, and utilizing experiential activities to apply knowledge in real-life situations.

On the other hand, user experience is defined as an episode that involves interactions between categories as follows: (1) an individual and an individual; (2) an individual and a service provider; and (3) a customer and a product provider resulting in a response [8]. Sheng and Teo [41] mentioned that user experience reflects an individual’s expectations and the stimuli that result from interactions between the categories noted previously [8]. The researchers also mentioned that experiences perceived by the students will be a complex feeling, and usually, it is difficult to distinguish between two different experiences. In this study, user experience refers to what is perceived by the students after being exposed to gamification in conservative classroom lectures of ethics education. Experience can be described in the form of feelings, emotions, behaviors, or mental state.

Finally, mobile app usability refers to a method of measuring a product’s ease of use [42]. Usability reflects the focus for every phase of design to ensure a mobile app is designed well, with usability in mind throughout the designing process [43]. For example, a mobile app that is designed well and fits the current needs of people would achieve high usability. However, app type and the location context may affect users’ mobile experiences [44], which eventually would contribute to poor usability. This study aimed to measure the usability of Ethoshunt as one of the constructs to ensure the app promotes fun and a meaningful learning experience of ethics education. Students are encouraged to explore Ethoshunt as a user-friendly tool that increases their chance of performing well in an ethics education course.

There are limited definitions and citations specifically related to correlations among positive emotions, ethical competency, user experience, and mobile app usability. This condition reflects the ways they can be integrated in gamification-related studies. The researchers found that the four constructs were established in gamification-related literature by many researchers, and it is critically important to measure mobile app usability to determine the usability of Ethoshunt. Thus, the four constructs fit the purpose of this study, which is primarily to present Ethoshunt as a gamification tool in ethics education. In this study, the researchers aimed to develop a mobile app involving game elements integrating the dynamics and mechanics of gamification. The mobile app Ethoshunt can store ethos points and ethos levels through hints sent by the ethics educator. Furthermore, this paper highlights the relationships between mobile app usability and positive emotions, ethical competency, and user experience, which were measured on a scale based on the developed mobile app. Finally, the researchers analyzed five open-ended questions to further explore participants’ personal experiences in using Ethoshunt.

## Methods

### Ethoshunt Development Process

The development of Ethoshunt is divided into four steps. The first step is the identification of the game mechanics and game dynamics of Ethoshunt. The second step is the definition of the components of the overall mobile app process module workflow. The third step is the presentation of how Ethoshunt functions. Finally, the fourth step is the identification of the learning flow through Ethoshunt integration.

### Participants

The participants were students enrolled in a counseling ethics education course at a public university in Malaysia. The students were all first semester undergraduate students who were diverse in terms of age, gender, race, and cultural identity. The participants were chosen based on the research purpose and consideration of the comprehensiveness of the resources available. Ethics education is a cut and dry course. The course is considered complex and covers a large number of topics. The students registered for the counseling ethics education course are required to read, digest, and memorize the contents included in the Malaysian Counselor Act 1998 (Act 580) and Counselors Code of Ethics [45]. They are also required to attend lectures that are conducted traditionally in a classroom setting and complete assignments given by their educator. Therefore, a change is needed to ensure that student motivation to learn ethics is sustained and to provide an engaging learning environment.

A total of 39 undergraduate counseling students participated in this pilot study. They were selected through cluster sampling, in which a particular batch of students was chosen as participants. The Ethoshunt development project was conducted throughout four full semesters, which is equivalent to 2 years. On the other hand, the ethics education course is offered once in a year, resulting in two batches or clusters of students for 2 continuous years. Each cluster consists of students studying in the following two programs: (1) Bachelor of Education in Guidance and Counseling with Honors and (2) Bachelor of Counseling with Honors. Therefore, one cluster of students was selected randomly as participants of this study, resulting in 39 counseling students. The population consisted of 84 participants.

### Data Analysis

Besides development of Ethoshunt, the researchers employed a mixed-methods research design to collect and analyze both quantitative and qualitative data gathered from a sample introduced to Ethoshunt. The researchers conducted a pilot study to assess the extent of positive emotions, ethical competency, user experience, and mobile app usability among undergraduate counseling students introduced to Ethoshunt and to capture their experiences of using Ethoshunt. The first stage of the pilot study involved survey analysis using Pearson correlation, and the purpose was to examine the relationships between two constructs as follows: (1) positive emotions and mobile app usability; (2) ethical competency and mobile app usability; and (3) user experience and mobile app usability. This was also called the quantitative phase.

The Gamification User Scale, a Malay language Likert-type scale ranging from 5 (strongly agree) to 1 (strongly disagree), was developed to measure positive emotions, ethical competency, user experience, and mobile app usability. Positive emotions, ethical competency, user experience, and mobile app usability are grouped into four sections, and the scale consists of 20 items. The first section consists of four items, and it measures students’ positive emotions regarding Ethoshunt. The second section consists of four items about mobile app usability. The third section consists of eight items on students’ ethical competency, and the final section involves four items that measure user experience. The scale has a high internal consistency (reliability, Cronbach α coefficient of .94). A panel of experts evaluated the content validity of the scale. Three experts, who are registered counselors with the Board of Counselors (Malaysia), evaluated the content validity of the scale in terms of comprehensiveness and clarity of the items. The examples of items for each construct are as follows:

- Positive emotions: (1) Ethoshunt is fun and (2) I am excited to learn while playing.
- Ethical competency: (1) Ethoshunt enhances my understanding in learning counseling ethics and (2) Ethoshunt assists me in application of counseling ethics education.
- User experience: (1) I am interested to learn using a mobile app in the future and (2) Ethoshunt makes me think about personal well-being.
- Mobile app usability: (1) A mobile app can make learning of counseling ethics more fun and (2) A mobile app can replace the traditional method of learning counseling ethics.

Another separate sheet was attached to the scale, and it involved the second stage of analysis, which is the qualitative phase. The separate sheet sought to collect students’ opinions based on their experiences using featured gamification elements. The researchers conducted analyses manually to understand students’ insights. An affinity diagram was used as a tool to analyze qualitative data by integrating the traditional qualitative simple data analysis method of using a pen and sticky notes. Opinions gathered from the students were transformed into an affinity diagram involving the following three steps: (1) capture; (2) group; and (3) label. The opinions from all participants were captured, grouped, and labeled to determine students’ insights regarding Ethoshunt as a gamification tool in ethics education. A total of five open-ended questions were included in the questionnaire, in addition to the scale (Multimedia Appendix 1). All opinions provided by the participants were coded using a traditional simple qualitative data analysis tool.

### Data Collection

The developed Ethoshunt app was used in parallel with conservative classroom lectures. It was not meant to replace the lectures, but rather complement them in a fun way. The ethics course instructor conducted the conservative classroom lectures for the first three topics. Thereafter, the instructor started the first set of hints, and the students followed the hints to get their ethos points, which eventually brought them to the next level of hints. The Ethoshunt was used until week 12 of the semester academic calendar. The ethics course instructor then rewarded the students who successfully managed to get to the next level up to the matured adult level, with treats, educational visits, or educational cash vouchers. Based on the developed Ethoshunt, the students’ positive emotions, ethical competency, user experience, and mobile app usability were assessed, and evaluations were conducted on a scale of 1 through 5. Students who used Ethoshunt indicated whether they strongly agree or strongly disagree with the items that reflect their perceived level of positive emotions, ethical competency, user experience, and mobile app usability. Furthermore, students responded to five open-ended questions concerning Ethoshunt. The surveys were distributed to the students, and they returned the surveys to the researchers after completion.

## Results

### Game Mechanics and Game Dynamics of Ethoshunt

Ethoshunt has been designed and created by the researchers through an understanding of game elements from traditional treasure hunt to aid learning, which is not seen as a serious game. Game mechanics refer to building blocks for gamifying offers, such as badges and scoring systems [35]. On the other hand, game dynamics describe the effects of game mechanics on students [35]. The game mechanics integrated in the mobile-based Ethoshunt trigger exploration in finding information relevant to the ethics course being taught, and there is a competition element as the game dynamic of Ethoshunt. Competition in Ethoshunt refers to a healthy game that creates collaboration among classmates or other game users in the long term. It involves a combination of game mechanics and motivational drivers to make Ethoshunt more fun and appealing to students. Levels, points, and rewards were also chosen as the game mechanics of Ethoshunt from other types of game mechanics, such as prizes, badges, and progress bars. In Ethoshunt, ethos levels eventually unlock the ethics course contents and ethos points increase the running numerical value of students’ work.

**Figure 1:**
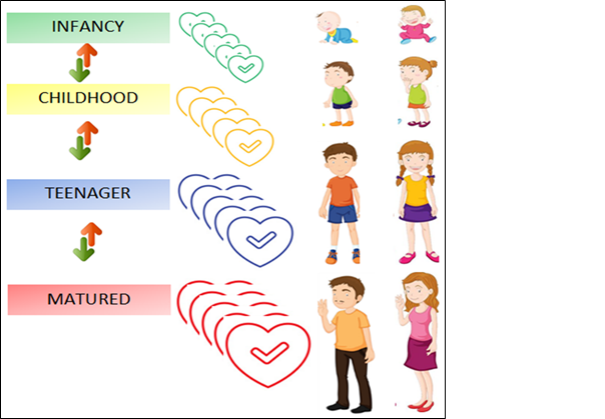
Game mechanics of Ethoshunt. The green arrow indicates advancement to the next level, and the red arrow indicates demotion to the previous level.

Figure 1 presents the game plan of Ethoshunt for introducing four levels of ethics acquisition, which include comprehension and application. All students can choose the gender (male or female). They start at the infancy level and progress to the childhood level, teenager level, and eventually matured adult level. If they fail to secure points at any level, they may be demoted to an earlier level. Students who have achieved the matured adult level are considered competent, and they are perceived to have gained good understanding, experience, and application of the ethics course content. Ultimately, students at the matured adult level will experience a sense of pride in learning the ethics education course.

### Ethoshunt System Architecture

**Figure 2:**
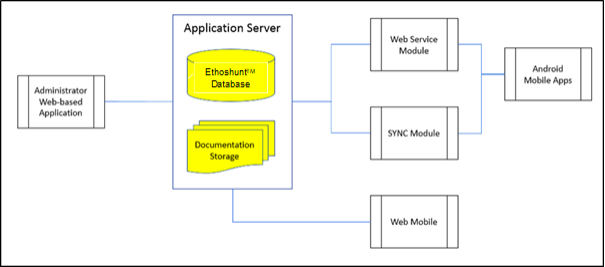
Ethoshunt module workflow.

Figure 2 illustrates the system architecture used in developing Ethoshunt. The system architecture was designed to handle three types of interfaces, including mobile web browser, desktop web browser, and Android mobile app. The administrators have a lot of information to view; hence, specifically for administrators, Ethoshunt is designed to be viewed using a desktop web browser. The system has been tested on the desktop web browsers Google Chrome (version 74.0.3729.131, official build, 64-bit; Google) on Windows 10 (Microsoft) and Safari (version 11.0.3; Apple) on Mac OS X El Capitan (version 10.11.6; Apple).

Users can use either a mobile web browser or the Android mobile app to access the system. The system has been tested on the mobile web browser Google Chrome (version 74.0.3729.157; Google), and the Android mobile app has been tested on Android Pie (version 9.0; Google).

All views from the mobile web browser, desktop web browser, and Android mobile app are connected to the application server that stores the Ethoshunt database. A unique architecture needs to be implemented for the Android mobile app, where a web service module and sync module need to be placed in between the interface and server to establish a compatible connection between the app and the server. This ensures a real-time update of the Android mobile app once the server has been updated using either a desktop or mobile web browser.

### Ethoshunt Functionality

**Figure 3:**
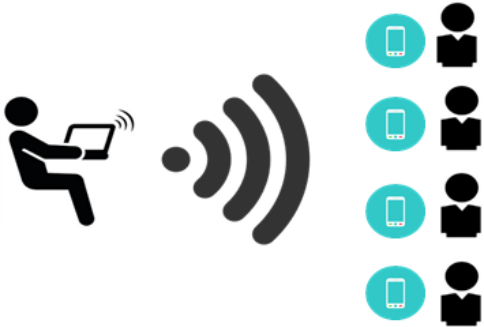
Hints sent by the ethics course instructor.

Game mechanics and game dynamics are two crucial components in a gamification-based mobile app. Hint is used as a game mechanic in Ethoshunt. The ethics educator sends hints relevant to the ethics course contents to the students (Figure 3). It can be at any time of the day. The dedicated Android app receives the hints automatically via message, and an alert is displayed. The types of hints can lead to two types of activities as follows: (1) hidden information in the virtual world that requires students to find internet stories, links, movies, notes, quotes, images, and sounds related to the topics being discussed, with guidance by the hints and (2) hidden information in the real world that requires students to find physical clues related to the topics being discussed, with guidance by the hints. Students then submit the answers to the ethics educator and are given points for correct answers.

In the context of this study, a total of 39 students used Ethoshunt. The ethics educator initially sent a hint to the students after completion of the first three topics. They responded to the hint by submitting the required format of answers to the ethics educator through Ethoshunt. Points were given for correct answers, and the process was repeated throughout the semester. The achievement of each student, which is the level of ethics acquisition, was determined at the end of the semester. Examples of hints sent to the counseling students and types of hints required by the ethics educator are as follows: (1) What makes counseling supervision effective? (text); (2) What are the ethical dilemmas encountered in counseling research? (animation); (3) Therapeutic counseling relationship (image); (4) The counselor as a person and professional (video); (5) What a counselor should do when his or her client reports suicidal ideation? (text); (6) Songs relevant to confidentiality (audio).

### Learning Flow Through Integrative Ethoshunt

**Figure 4:**
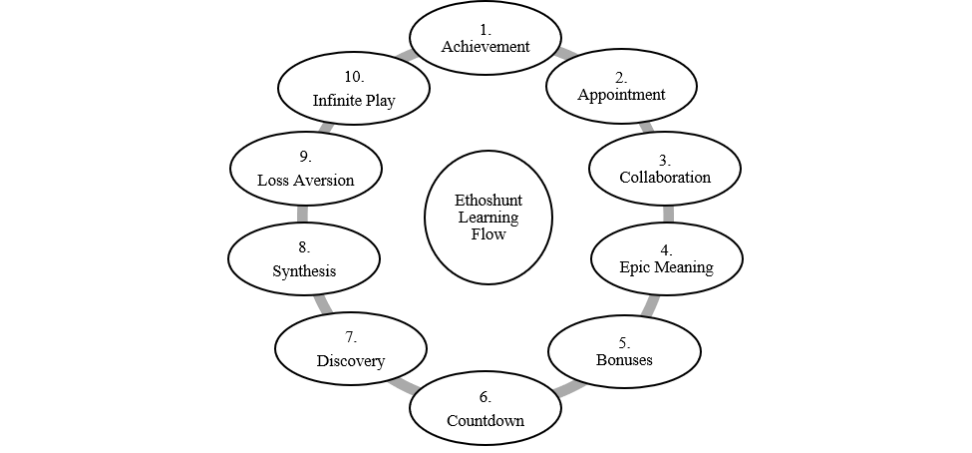
Learning flow through Ethoshunt.

Students who use Ethoshunt will be able to take part in the learning process through a meaningful educational flow. Through this flow, students develop their competence in ethics education and meaningfully reflect on what has been taught by the ethics educator prior to making their own revisions. Furthermore, this flow strengthens the process of conveying ethics course contents using gamification. Figure 4 shows the learning flow that every student will experience. First, achievement enables students to earn public recognition (learning peers and educator) for completing the tasks given. Second, students will check on their appointments, where they need to check in to receive new hints from their ethics educator. Third, students will collaborate and work with other learning peers to accomplish learning goals. Fourth, students are encouraged to work on the epic meaning, where they are expected to work to achieve excellent learning outcomes. Thereafter, students will receive unexpected rewards or bonuses for their achievements, and this is to motivate students to move forward and set countdowns. Through countdowns, students will tackle challenges in a limited amount of time. The seventh component in the flow is discovery. Students will navigate through the learning space and uncover pockets of knowledge. Students will then learn to synthesize the knowledge they have gained and work continuously on challenges that require multiple skills to solve. The ninth component is loss aversion, where students play to avoid losing what they have already gained, and finally, students will go through infinite play, where they continuously learn until becoming experts in the field. Students who are active throughout the learning process involving Ethoshunt will achieve unexpected positive outcomes.

### Survey Analysis

**Figure 5:**
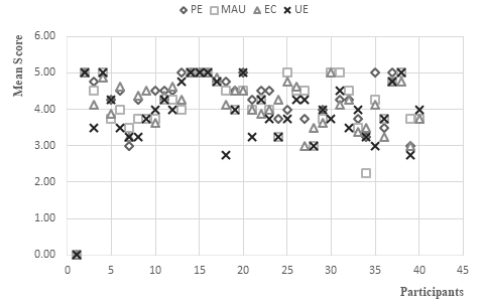
Distribution of mean scores. EC: ethical competency; MAU: mobile app usability; PE: positive emotions; UE: user experience.

The mean scores of positive emotions, ethical competency, user experience, and mobile app usability were calculated. The mean scores ranged from 2.2 to 5.0, which indicates that most of the participants’ selections on the scale fell between 2 (disagree) and 5 (strongly agree). Figure 5 shows the distribution of the mean scores among the participants. The distribution pattern suggested that there were relevant relationships among positive emotions, ethical competency, user experience, and mobile app usability.

For the first stage of analysis (Table 1), Pearson correlation indicated that positive emotions, ethical competency, and user experience were significantly related to mobile app usability. Positive emotions were positively related to mobile app usability (*r*=0.744, *P*<.001). There were also significant positive relationships between ethical competency and mobile app usability (*r*=0.686, *P*<.001) and user experience and mobile app usability (*r*=0.614, *P*<.001).

**Table 1:** Correlation coefficients for positive emotions, ethical competency, and user experience.

Variable/relationship	Correlation with mobile app usability (*r*)
Positive emotions	0.744
Ethical competency	0.686
User experience	0.614

Thereafter, Guildford rule of thumb was used to determine the strength of the relationships. Based on the rule of thumb (Multimedia Appendix 2), positive emotions had high correlation, followed by ethical competency and user experience, which had moderate correlation with mobile app usability.

### Insights From Participants

For the second stage of analysis and referring to question one in Multimedia Appendix 1, it was found that 74% (29/39) of students had a better understanding of ethics education. They mentioned that their knowledge about ethics expanded, and it was their most meaningful experience. However, the opinions of 26% (10/39) of students were focused specifically on the ethics educator, ethics course, and classroom, instead of delivering perceptions about their most meaningful experiences in learning ethics.

For the second question, 36% (14/39) of students mentioned that the opportunity given to them to present in the class and the ethics educator’s sharing of own experiences in ethics education were the most helpful experiences in the ethics class. They were able to make their own reflections based on the support provided by the ethics educator and were able to share their reflections with their classmates. One of the students mentioned that knowledge gained through the technology-based education tool was the most helpful experience, as the student could learn while enhancing the understanding of ethics. The remaining students stated that the activities conducted in the ethics class and the support from the ethics educator were the most helpful experiences in learning ethics.

In responding to what can be improved in the ethics education class, 26% (10/39) of students were expecting technology-based elements in learning ethics and 74% (29/39) of students focused on traditional teaching methods. For the fourth question, 38% (11/39) of students stated that the technology-mediated ethics education class can be improved by providing high-speed internet facilities in the classroom, extending the duration of the ethics class, and including more game elements in the mobile app. The rest of the students suggested frequent use of game-based tools in the classroom, as well as an attractive, colorful, and user-friendly mobile app. Finally, for the last question, 59% (23/39) of students wanted to see improvement in the elements of Ethoshunt. They suggested more game elements, more challenging questions, and more button functions, as well as the inclusion of animation with motions, attractive graphics, and ethics notes in games to improve ethics education classes that use Ethoshunt. The remaining students commented on the external factors of Ethoshunt, such as accessing Ethoshunt offline and internet speed.

## Discussion

### Principal Findings

In this study, the researchers found relationships between mobile app usability and positive emotions, ethical competency, and user experience. In addition, the researchers gathered new insights to be incorporated as ideas of gamification in ethics education. It is interesting to note that most of the students had meaningful experiences in learning ethics, and they considered Ethoshunt as a good mobile app with room for improvement.

The results of the survey analysis showed that there were relevant relationships between positive emotions and mobile app usability, ethical competency and mobile app usability, and user experience and mobile app usability. The highest correlation with mobile app usability was noted for positive emotions, followed by ethical competency and user experience. The results are in line with findings from other studies, which emphasize positive emotions as the effect of gamification techniques [35,36]. Blohm and Lelmelster [6] mentioned that gamification promotes behavior changes that could be the result of positive emotions exhibited after being exposed to gamification elements. Students who have experienced positive emotions as a result of using gamification in ethics education would most probably support mobile app usability. They would consider a gamification-based mobile app as a useful tool that can assist them in learning ethics. On the other hand, ethical competency and user experience recorded moderate relationships with mobile app usability. It can be seen that ethical competency and user experience do not greatly alter the idea of using gamification in ethics education. Although Ethoshunt was used to enhance the understanding of ethics education, students still perceived that there was not much difference in their ethical competency after using Ethoshunt. Their experience of using Ethoshunt was also almost similar to what they experience with traditional teaching and learning methods. Their positive emotions flourished more compared with ethical competency and user experience, which indicates the great impact of using gamification in ethics education. The findings reflect that positive emotions were associated with the usability of Ethoshunt. The primary objective of developing Ethoshunt was to improve students’ ethical competency. However, the findings showed that students prefer a fun, contented, and interesting learning environment to learn ethics education. This would eventually help them to invest more time in exploring Ethoshunt and learn ethics education simultaneously.

The second stage of analysis that involved qualitative data reported various comments or feedback from students. Most of the students experienced fun and joy in learning ethics. They preferred more games in ethics education. However, they suggested providing high-speed internet and more time to explore Ethoshunt. All students participating in the study owned mobile phones and were able to access mobile data. However, the speed of the internet varies depending on multiple factors, such as network, mobile processor, and type of mobile phone. In addition, the design of the mobile app affects usability. A mobile app that is designed well will definitely attract users and increase the chance of improving usability [43].

### Limitations and Future Research

Past literature reviews relevant to gamification that can explain positive emotions, ethical competency, user experience, and mobile app usability are very limited. This restricted the researchers in providing arguments that could support the research findings. Ethoshunt requires good internet connectivity, which is a constraint for some students who do not have access to smartphones and the internet. As for gamification itself, the newly developed online-based mobile app Ethoshunt can be used for all ethics-based courses, which can be expanded in the future to benefit other disciplines, such as ethics in engineering, ethics in medicine, ethics in law, ethics in business and marketing, ethics in technology, etc. In the future, a larger sample size can be used to broaden the perspectives of gamification, and assessments should be conducted in large-scale actual research.

### Conclusion

Ethics education is one of the most important areas of knowledge acquisition in any profession. It is the responsibility of ethics educators to prepare students to learn, understand, experience, and apply ethics education to their personal and professional work. Lack of ethics knowledge and self-care awareness can contribute to poor ethical decision-making ability. Therefore, it is necessary to implement new strategies in teaching and learning to enhance the understanding of ethics education among students. The integration of gamification elements, integration of the use of game dynamics and game mechanics, and implementation of gamification deserve greater attention. Ethoshunt, a gamification-based mobile app, is one of the tools that can be integrated in teaching and learning ethics, as it enables creative and intuitive skills, which may encourage students to learn ethics more attractively and effectively. Furthermore, the nature of ethics education is complicated, and it is quite challenging to comprehensively teach ethics education in a semester. Thus, ethics educators may use Ethoshunt (although it requires improvement) to disseminate and apply ethics-related knowledge while making learning enjoyable.

## Appendix

Multimedia Appendix 1 Open-ended questions regarding Ethoshunt.

Multimedia Appendix 2 Guilford rule of thumb illustrating the strength of relationships.
