# Supplementary material for: Assessing Ethoshunt as a Gamification-Based Mobile App in Ethics Education: Pilot Mixed-Methods Study
Source: JMIR Serious Games. 2020 Aug 10;8(3):e18247. doi: 10.2196/18247 (PMC7445620; doi:10.2196/18247)
Supplement: Multimedia Appendix 1 [file games_v8i3e18247_app1.docx]

**Multimedia Appendix 1.** Open ended questions regarding Ethoshunt^TM^

1. What is your most meaningful experience in learning ethics?

2. What is your most helpful experience in learning ethics?

3. What can be improved in ethics education lectures?

4. What can be improved in ethics education lectures that uses mobile application?

5. What can be improved in ethics education lectures that uses Ethoshunt^TM^?
