# Supplementary material for: Assessing Ethoshunt as a Gamification-Based Mobile App in Ethics Education: Pilot Mixed-Methods Study
Source: JMIR Serious Games. 2020 Aug 10;8(3):e18247. doi: 10.2196/18247 (PMC7445620; doi:10.2196/18247)
Supplement: Multimedia Appendix 2 [file games_v8i3e18247_app2.docx]

**Multimedia Appendix 2.** Guilford’s rule of thumb illustrating strength of relationships

| *r* | Strength of relationship |
| --- | --- |
| < .20 | Almost negligible |
| .20 - .40 | Low |
| .40 - .70 | Moderation |
| .70 - .90 | High |
| > .90 | Very high |
